# Comprehensive characterization of immunoglobulin gene rearrangements in patients with chronic lymphocytic leukaemia

**DOI:** 10.1111/jcmm.12215

**Published:** 2014-04-11

**Authors:** Céline René, Nathalie Prat, Audrey Thuizat, Mélanie Broctawik, Odile Avinens, Jean-François Eliaou

**Affiliations:** a Department of Immunology, CHRU de Montpellier, University Hospital Saint-EloiMontpellier, France; b Faculté de Médecine, University of Montpellier 1Montpellier, France

**Keywords:** immunoglobulin, somatic hypermutation, CLL, ageing, AID, CDR3

## Abstract

Previous studies have suggested a geographical pattern of immunoglobulin rearrangement in chronic lymphocytic leukaemia (CLL), which could be as a result of a genetic background or an environmental antigen. However, the characteristics of Ig rearrangements in the population from the South of France have not yet been established. Here, we studied CLL B-cell repertoire and mutational pattern in a Southern French cohort of patients using an in-house protocol for whole sequencing of the rearranged immunoglobulin heavy-chain genes. Described biased usage of variable, diversity and joining genes between the mutated and unmutated groups was found in our population. However, variable gene frequencies are more in accordance with those observed in the Mediterranean patients. We found that the third complementary-determining region (CDR) length was higher in unmutated sequences, because of bias in the diversity and joining genes usage and not due to the N diversity. Mutations found in CLL followed the features of canonical somatic hypermutation mechanism: preference of targeting for activation-induced cytidine deaminase and polymerase motifs, base change bias for transitions and more replacement mutations occurring in CDRs than in framework regions. Surprisingly, localization of activation-induced cytidine deaminase motifs onto the variable gene showed a preference for framework regions. The study of the characteristics at the age of diagnosis showed no difference in clinical outcome, but suggested a tendency of increased replacement and transition-over-transversion mutations and a longer third CDR length in older patients.

## Introduction

Chronic lymphocytic leukaemia (CLL) is the most common leukaemia, affecting adults in Western countries. The clinical outcome of CLL is very variable, ranging from patients having an aggressive malignancy to others having a slow, non-progressive disease. The determination of the somatic mutational status of the rearranged immunoglobulin IGHV genes has emerged as a strong prognostic factor to stratify patients in clinical trials [1–4]. In particular, patients with 2% or greater mutations on IGHV genes (so-called ‘mutated’) have a better prognosis than patients with less than 2% mutations (so-called ‘unmutated’).

A biased usage of IGHV genes has been described in CLL cells with a preference for IGHV1, IGHV3 and IGHV4 genes, but with a different repartition between mutated and unmutated groups [5–9]. Particularly, IGHV1-69 segment was associated with unmutated status, whereas IGHV3-23 and IGHV4-34 segments were found in patients with a mutated status [5,8,9]. Among the IGHD genes, IGHD3 and, in particular, IGHD3-3 was largely overrepresented and associated with unmutated status [2,5,8,9]. Concerning the IGHJ genes, IGHJ4 was preferentially used in the mutated group, whereas IGHJ6 was mainly found in the unmutated group [2,5,7–9]. In addition, the comparison of several studies conducted in different geographical regions led to the observation of disparities in IGH gene frequencies [8,10].

Previous studies showed that third complementary-determining region (CDR3) was longer in the unmutated group than in the mutated group [5,8,9,11]. A shorter CDR3 was observed in rearrangements using IGHV3 family compared with other IGHV families [5]. Considering the most frequently used IGHV genes, the average size of CDR3 in B-cell receptor (BCR) containing the IGHV1-69 gene was longer than for other IGHV segments [8,12,13]. A significant longer CDR3 was observed in BCR including the IGHJ6 gene compared with IGHJ3 and IGHJ4 [8,11,13,14]. Moreover, Stamatopoulos *et al*. demonstrated that the IGH genes were rearranged in a non-random manner and showed the existence of BCR stereotypes [11].

The study of the mutational pattern in the IGHV gene in CLL patients showed characteristics of somatic hypermutation (SHM), including less replacement (R) mutations in framework (FR) compared with CDRs of IGHV genes, excess of transitions over transversions and mutations targeting specific nucleotides or nucleotide motifs specific of AID (RGYW) and polymerases (WA) [8,15,16].

Studying the IGHV3-21 gene, Ghia *et al*. suggested a geographical pattern of Ig rearrangement in CLL [10], with a difference between the Mediterranean and Scandinavian populations. The purpose of this study was to determine the characteristics of Ig rearrangements in our population from the South of France and to compare them with previously published data. Using an in-house multiplex protocol with IGHV-Leader primers to efficiently amplify and sequence the entire IGHV gene, we analysed the biased usage of the IGHV, IGHD and IGHJ genes. Furthermore, we compared the CDR3 length between mutated and unmutated groups and analysed the contribution of each CDR3 components. We also focused on the nucleotide changes in patients with mutated IGHV gene to evaluate whether the mutational pattern was compatible with SHM. To this aim, we analysed transition bias, silent versus replacement mutation ratio, and localization of the RGYW and WA motifs. Finally, in absence of previous study, we determined whether there are differences in IGHV, IGHD or IGHJ usage, CDR3 length, accumulation of mutations and mutation characteristics in CLLs cells related to the age of diagnosis.

## Materials and methods

### CLL patients and DNA

Seventy-four CLL patients were followed up at the University Hospital of Montpellier between 1997 and 2011. For study according to the age at diagnosis, three categories were empirically determined by decade of age. Genomic DNAs were extracted using QIAmp DNA Blood Mini Kit (Qiagen®, Courtaboeuf, France) according to the supplier's protocol. Patients signed a written consent for analysis.

### Multiplex PCR conditions and genescan analysis

Two protocols were compared to assess the clonal pattern of each patient. The first protocol, called ‘IGHV-Leader’, used a mix of 5′ primers specific for each leader sequence located 150 bp upstream of the IGHV region of the IGHV1 to IGHV6 families together with the 3′ BIOMED consensus JH-FAM primer (listed in Table1). The other, called ‘BIOMED2-FR1’, used the mix of 5′ FR1 primers and the JH-FAM consensus as described by the BIOMED2 protocol [17].

**Table 1:** IGHV-Leader family and JH consensus primers

IGHV-leader family primers	Sequences
IGHV1	5′-CCATGGACTGGACCTGGA-3′
IGHV2	5′-ATGGACATACTTTGTTCCAC-3′
IGHV3	5′-CCATGGAGTTTGGGCTGAGC-3′
IGHV4	5′-ATGAAACACCTGTGGTTCTT-3′
IGHV5	5′-ATGGGGTCAACCGCCATCCT-3′
IGHV6	5′-ATGTCTGTCTCCTTCCTCAT-3′
JH-FAM consensus primers	5′-CTTACCTGAGGAGACGGTGACC-3′

For the IGHV-Leader procedure, the VDJ rearrangement amplification was performed in a 25 μl final volume, according to the manufacturer's protocol (Multiplex PCR kit; Qiagen®).Thirty-seven cycles of amplification were performed under the following conditions: 30 sec. at 95°C, 90 sec. at 57°C, 90 sec. at 72°C with an initial denaturation/activation step at 95°C during 15 min. and a final extension step at 72°C for 10 min.

For the standardized BIOMED-2 multiplex protocol, PCR was performed as previously described [17] in a final volume of 50 μl.

Two microlitres of the PCR product was run on a sequencer.

### PCR for VH assignment

For both protocols, PCR products were purified according to the manufacturer's protocol (QIAQuick MinElute PCR Purification Kit; Qiagen®). Then, six PCR reactions were performed with each of six sense family-specific primers (for the IGHV-Leader protocol) or FR1 primers (for the BIOMED2-FR1 protocol) in combination with an antisense JH primer. PCR conditions were identical to those of multiplex PCR. For each PCR, control was performed on a 2.5% agarose gel.

### Immunoglobulin rearrangement sequencing

After a first purification step using ExoSAP-IT® kit (GE Healthcare, Velizy-Villacoublay, France) according to the supplier's protocol, the PCR products were sequenced in both directions using the appropriate sense and antisense primers. Each amplification mix included: 0.1 μM of primers (IGHV-Leader, FR1 or JH); 2 μl of Big Dye Master Mix 10× (Big Dye Terminator v3.1 Cycle Sequencing kit; Applied Biosystem®, Saint Aubin, France); 4 μl Buffer 5× qsp 15 μL. This mix was added to 6 μl of PCR products. This PCR was performed in 25 cycles (10 sec. at 96°C, 5 sec. at 50°C and 4 min. at 60 °C). Then, PCR products were purified on Sephadex® plate (GE HealthCare) and run in an Applied Biosystem® Sequencer 3130XL.

### Analysis of IGHV-D-J sequence

The sequences obtained from sense and antisense primers were first aligned and then analysed in two databases: IMGT/V-QUEST tool (International ImMunoGeneTics information system, M-P Lefranc, University of Montpellier, CNRS, France; http://www.imgt.org/IMGT_vquest/) and IgBLAST software (National Center of Biotechnology Information, National Institutes of Health, Bethesda, MD, USA; http://www.ncbi.nlm.nih.gov/igblast/). The results were reported following the IMGT format. We considered only productive sequences.

To analyse the mutations in the IGHV gene, we used the IMGT/V-QUEST tool. CDR and FR regions were as defined by IMGT-V-QUEST. The per cent of homology was calculated by counting the number of nucleotide differences between the 5′ end of FR1 and the 3′ end of the FR3 of the VH sequence. We considered as mutated an IGHV gene sequence presenting more than 2% sequence alterations when compared with the published germline sequence. Patients with a percentage of mutations of more than 2% were included in the mutated group [18]. In mutated sequences, Lossos formula was used to determine if antigen selection occurred [19]. The sequences were also analysed for CDR3 length, CDR3 motifs, RGWY and WA motifs, and mutation localization using IMGT/V-QUEST tool.

To calculate the probability that mutation occurring in AID (RGWY/WRCY) or polymerases (WA/TW) motifs was not because of hazard, we estimated the expected frequency of these motifs and compared it with the observed frequency. The expected frequency of mutations targeting the RGYW/WRCY or WA/TW was obtained by estimating the expected number of mutations located in a RGYW/WRCY or WA/TW motif if the mutation repartition was random and by taking into account the length of the motif and the number of each motif in a given IGHV sequence. The observed frequency of mutations located in the motifs of interest was computed by taking into account the number of mutations located in the motifs and the total number of nucleotides.

### Statistical analysis

The chi-squared statistic was used for categorical and the Student's test or Kruskal–Wallis for continuous variables. *P* < 0.05 was considered as significant. When the overall test was significant, pairwise analyses of each group were performed. To estimate the correlations between quantitative parameters, we used the nonparametric Spearman's rank order coefficient.

## Results

### Entire IGHV sequencing using IGHV-Leader primers gives the same detection rate and mutational status as partial IGHV sequencing using BIOMED2-FR1 primers

To determine the IGHV mutational status, the ERIC group (European Research Initiative on CLL) recommends choosing between two sets of primers: the IGHV-Leader or the BIOMED2-FR1 primers [20]. IGHV-Leader primers targeted the Leader sequence located 150 bp upstream of the IGHV gene. Compared to the BIOMED2-FR1, they allow the whole sequencing of the IGHV region and thereby a precise definition of the percentage of identity to the closest germline gene. As drawbacks, they are known to be less efficient for detecting a clonal pattern than the standardized BIOMED2-primers.

To compare our in-house protocol for IGHV gene complete sequencing using IGHV-Leaders primers with the standardized BIOMED2-FR1 protocol, we studied 74 CLL patients. Briefly, the DNA of each patient was amplified with both protocols and analysed by Genescan® (Applied Biosystem, Saint-Aubin, France) after migration on a sequencer. Although the sensitivity of detection (*i.e*. the capacity to detect a clonal population when it is present) was the same with both protocols (85%), some differences could be observed among the DNA samples. In one situation, the IGHV-Leader primers were able to detect, in three patients, a clonal rearrangement, which was not detected using the IGHV-FR1 primers. Analysis of the sequence from these patients showed numerous mutations in the IGHV-FR1 primer hybridization region. In another situation, three patients showed a monoclonal pattern only using the IGHV-FR1 primers. Overall, the combination of the two methods allowed us to slightly improve the sensitivity for detecting clonal rearrangements (93%).

**Figure 1:**
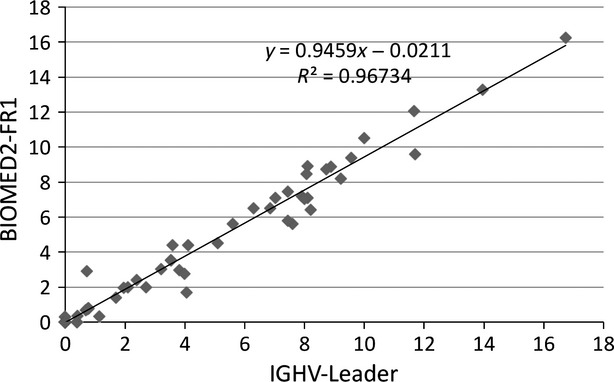
Comparison of mutational rate according to the set of primers used. The IGVH mutation percentage was determined by either an entire IGVH sequencing with IGHV-Leader primers (*x* axes) or partial sequencing with IGHV-FR1 primers (*y* axes). The percentage was obtained by dividing the number of mutations by the length (in nucleotides) of each region. One plot corresponds to one patient. Comparisons were performed in 64 patients by a Spearman's correlation test (correlation coefficient *R*^2^ = 0.97, *P* < 0.0001).

To assess the reliability of the two methods for determining the mutational status, we compared data obtained with both primer sets (Fig.1) and showed a strong correlation (*R*^2^ = 0.97, *P* < 0.0001). As both protocols give the same results, we used the whole sequences obtained with IGHV-Leaders primers for further analyses.

### Usage of IGHV, IGHD and IGHJ genes is biased and differs between mutated and unmutated status

Next, we analysed the IGHV, IGHD and IGHJ genes usage according to the mutational status of the patients. 57.6% of patients (38/66) exhibited a mutated IGHV sequence, and 42.4% of patients (28/66) had an unmutated status. Among the unmutated group, 10 patients presented 100% homology with the germline sequence.

**Figure 2:**
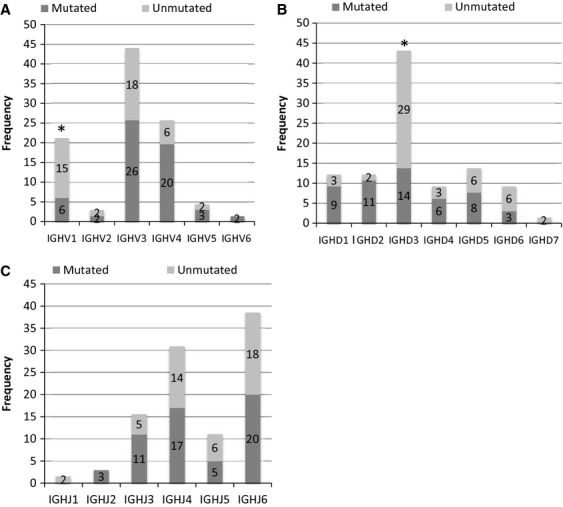
IGHV, IGHD and IGHJ genes usage in rearranged B-cell receptor in mutated and unmutated groups of chronic lymphocytic leukaemia (CLL) patients. (A) Comparison of IGHV family gene usage between CLL mutated patients (dark grey bars) and unmutated patients (grey bars). Asterisks indicate significant difference (*P* < 0.05). (B) Comparison of IGHD family gene usage between CLL mutated patients (dark grey bars) and unmutated patients (grey bars). Asterisks indicate significant difference (*P* < 0.05). (C) Comparison of IGHJ family gene usage between CLL mutated patients (dark grey bars) and unmutated patients (grey bars).

The most commonly used IGHV genes were IGHV3 (*n* = 29, 44% of total patients), IGHV4 (*n* = 17, 26%) and IGHV1 (*n* = 14, 21%). Analysis of the immunoglobulin gene usage revealed a statistically significant (*P* < 0.05) preferential usage of IGHV1 family in unmutated group *versus* mutated group (*n* = 10, 71% *versus n* = 4, 29% of IGHV1 total cases respectively). On the contrary, for IGHV4 family, 13 of 17 patients (77% of IGHV4 total patients) belong to the mutated group (Fig.2A). When regarding the frequency of individual V segments, the IGHV1-69 segment was only used in the unmutated configuration, in accordance with prior studies [8,10,21]. Eighty-three per cent of patients (5/6) presented the IGHV1-69 gene associated with the IGHD3-3 gene and a IGHJ6 segment. Conversely, IGHV4-34 segment was only found in the mutated group.

Considering the IGHD genes, the rearrangements found in CLL patients predominantly used IGHD3 family (42%), in particular IGHD3-22, IGHD3-10 and IGHD3-3. In the unmutated group, a preferential usage of IGHD3 family (*P* < 0.05) was observed (Fig.2B). Among this gene family, the IGHD3-3 gene and IGHD3-22 were more frequently found in a germline configuration (7/8 cases and 6/8 cases respectively). On the other hand, the patients with mutated IGHV gene presented more rearrangements including IGHD1 and IGHD2 genes.

Concerning the IGHJ gene usage, IGHJ6 and IGHJ4 segments were observed in 69% of patients (*n* = 25 and *n* = 20, respectively; Fig.2C). Usage of IGHJ segments did not differ between mutated and unmutated groups.

### Higher CDR3 length in unmutated sequences is because of bias in the diversity and joining genes usage, but due to not the N diversity

The size of the CDR3 resulted from the IGHV, IGHD, IGHJ gene selected and the number of P and N nucleotides added. We examined CDR3 length in relation to the IGHV gene used into the rearranged BCR for all the patients (Table2). We found that the average CDR3 length was identical for IGHV3 or IGHV4 selected gene (50 and 53 base pairs, respectively) while it was longer for IGHV1 gene (64 base pairs, *P* < 0.01). The CDR3 length was not significantly different between the IGHD gene families (Table2). According to prior studies [8,11,13,14], a significant longer CDR3 in the BCR rearranged with IGHJ6 segment compared with IGHJ3 and IGHJ4 was observed (61 *versus* 52.9 and 49.5, respectively, *P* < 0.01; Table2).

**Table 2:** CDR3 length in mutated and unmutated group according to the IGHV, IGHD and IGHJ family genes

		Mutated group (mean length in bp)	Unmutated group (mean length in bp)	All the patients (mean length in bp)	*P* value (mutated *versus* unmutated)
IGHV	IGHV1	47.25	71	63.7*	<0.01
	IGHV2	51	Undetermined	51	Undetermined
	IGHV3	49.9	51.4	50.4	NS
	IGHV4	50.3	57.6	52.9	NS
	IGHV5	46.5	Undetermined	46.5	Undetermined
	IGHV6	Undetermined	Undetermined	Undetermined	Undetermined
IGHD	IGHD1	48.6	49	49.7	NS
	IGHD2	56	61.5	55.6	NS
	IGHD3	48	69.5	61.2	<0.001
	IGHD4	55	48	54	Undetermined
	IGHD5	42	33	39	Undetermined
	IGHD6	44	57	50.5	<0.05
	IGHD7	24	Undetermined	24	Undetermined
IGHJ	IGHJ1	Undetermined	48	48	Undetermined
	IGHJ2	36	Undetermined	36	Undetermined
	IGHJ3	51.9	60	52.9	Undetermined
	IGHJ4	47.3	54	49.5	NS
	IGHJ5	46.5	60	51	Undetermined
	IGHJ6	53.3	66.3	61*	<0.01

* CDR3 length statistically different from the others family genes (*P* < 0.05).

Comparing CDR3 length according to the mutational status of the patients, significantly longer CDR3s were observed in unmutated *versus* mutated sequences (Table2, 61.3 *versus* 49.5 base pairs; *P* < 0.05). To evaluate whether a particular IGHV, IGHD or IGHJ gene family was associated with this disparity, we analysed the mean of CDR3 length in each gene family in the two groups. The results showed a significant longer CDR3 region in the unmutated group compared with the mutated group only in rearrangements bearing a IGHV1 (Table2; 71 *versus* 47.3 base pairs, respectively, *P* < 0.01), or a IGHD3 (Table2; 69.5 *versus* 48, respectively, *P* < 0.001), or a IGHJ6 gene family (Table2; 66.3 *versus* 53.3, respectively, *P* < 0.01). In other cases, there was no significant difference in CDR3 length between mutated and unmutated status. Next, to identify the elements responsible for this disparity, we determined the number of nucleotides in the CDR3 provided by the IGHV, IGHD, IGHJ, P and N elements in the mutated and the unmutated group (Table3). The results showed a significant higher length of the IGHD and IGHJ gene segments included in the CDR3 region in the unmutated group than in the mutated group (17.4 *versus* 12 base pairs for IGHD genes, *P* < 0.01 and 21.4 *versus* 14.3 for IGHJ genes, *P* < 0.001 respectively). Moreover, IGHD3-3 and IGHD3-22, more frequently found in a germline configuration, contributed to CDR3 length for 23.1 nucleotides *versus* 13.5 nucleotides for other IGHD3 segments (*P* < 0.001). The IGHJ6 segments contributed to more nucleotides in CDR3 than other IGHJ segments (24.7 nucleotides *versus* 12.4, *P* < 0.0001). Other elements did not significantly contribute to the CDR3 length disparity between unmutated and mutated groups. Interestingly, the percentage of mutations and CDR3 length were inversely correlated (*r* = −0.38, *P* < 0.05).

**Table 3:** Comparison of nucleotides length of CDR3 components in mutated and unmutated group

Components (mean length in bp)	Mutated	Unmutated	*P* value (mutated *versus* unmutated)
Total CDR3	49.5	61.3	<0.01
IGHV segment	9.2	9.7	NS
IGHD segment	12	17.4	<0.01
IGHJ segment	14.3	21.4	<0.001
P nucleotides	0.17	0.33	NS
N nucleotides in 5′ of IGHD	6	7	NS
N nucleotides in 3′ of IGHD	7.7	5.4	NS

### Mutational pattern of the IGHV gene shows evidence of antigen-driven selection

**Figure 3:**
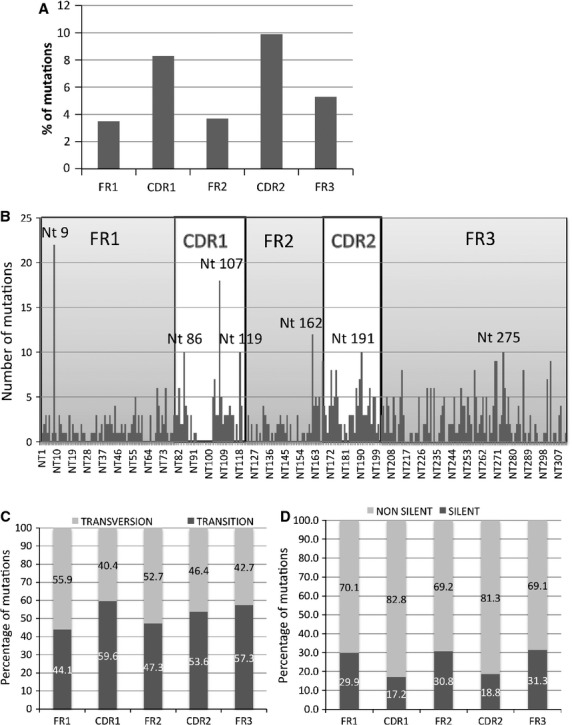
Localization of mutation along the IGHV gene. (A) Frequency of mutation in the FR and complementary-determining region (CDR). The frequency was calculated by dividing the number of mutations by the length (in nucleotides) of each region. (B) Mapping of the mutations in the IGHV gene. Each bar corresponds to the number of mutations found at a given position. Nt was used to abbreviate nucleotide. The nucleotide positions with mutations found in at least 10 patients are indicated. (C) Distribution of transversion and transition mutations in FR and CDR regions. The results are expressed in percentage of mutations inducing a transversion (grey bars) or a transition (dark grey bars). (D) Distribution of silent and replacement (non-silent) mutations in the FR and CDR regions. The results are expressed in percentage of mutations inducing an amino acid change (grey bars) or silent mutation (dark grey bars).

Considering only mutated sequences whatever the mutational status of the patients, the frequency of mutations was assessed for each FR and CDR1, CDR2 regions. Results showed a higher mutation frequency in the CDR regions than in FR regions (Fig.3A). However, it was interesting to note that FR3 presented more mutations than FR1 and FR2 regions. Twenty-two of 38 (58%) patients presented at least one mutation among the following positions: positions 9 (FR1), 86, 107, 119 (CDR1), 162 (FR2), 191 (CDR2) and 275 (FR3) (Fig.3B). While mutations were distributed along the gene, no patient presented mutation from the position 94 to 102 in the CDR1.

Next, we evaluated the characteristics of nucleotide changes. Taking into account all the mutations, we observed a preference for transition (purine to purine or pyrimidine to pyrimidine)-over-transversion (purine to pyrimidine or pyrimidine to purine) mutation within the IGHV gene (53.4% *versus* 46.6% respectively). Except for FR3, we observed significantly more transitions in the CDR1 and CDR2 than in the FR (56.4% *versus* 45.4%, *P* < 0.05; Fig.3C). It was interesting to note that the FR3 contained the same proportion of transitions than in CDRs.

Then, we investigated whether mutation induced an amino acid change. Overall, 71.4% of mutations induced an amino acid change. Analysis of the distribution of R (Replacement) and S (Silent) mutations showed a significant clustering of R mutations in the CDR regions and conservation of the amino acid sequence in the FRs (*P* < 0.001; Fig.3D). In the mutated sequences, Lossos formula was used to determine if antigen selection occurred [19]. A higher frequency of R mutations and a lower frequency of S mutations in the CDRs compared with the FRs suggest an antigen selection process. For a given IGHV gene, evidence of antigen-driven selection was likely if the applied statistical model yielded a significant result (*P* < 0.05). Using this approach, we found that 7/36 of patients (19%) had mutated rearrangements with only a scarcity of R mutations in FR, 6/36 of patients (17%) with only a significant excess of R mutations in CDR and 9/36 (25%) with both scarcity of R mutations in FR and excess in CDR. Taken together, 61% of patients had rearrangements with evidence of antigen-driven selection.

### FR regions display more mutations in AID or polymerases motifs than CDR

**Figure 4:**
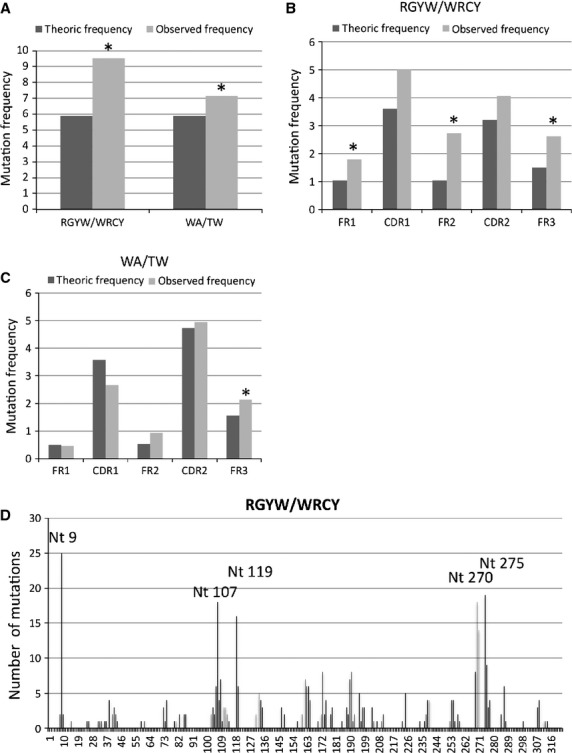
Mutation localization in AID (RGYW/WRCY) and polymerase (WA/TW) motifs. (A) Comparison between expected and observed mutation frequencies targeting the RGYW/WRCY or the WA/TW motifs. The expected frequency of mutations targeting the RGYW/WRCY or WA/TW (dark grey bars) was obtained by estimating the expected number of mutations located in a RGYW/WRCY or WA/TW motif if the mutation repartition was random and by taking into account the length of the motif and the number of each motif in a given IGHV sequence. The observed frequency of mutations located in the motifs of interest (grey bars) was computed by taking into account the number of mutations located in the motifs and the total number of nucleotides. Differences between expected and observed values were assessed by Chi-squared test. Asterisks indicate significant difference (*P* < 0.001). (B) Comparison between expected and observed mutation frequencies targeting the RGYW/WRCY motifs in the FR and complementary-determining region (CDR) regions. Analysis of expected (dark grey bars) and observed frequencies (grey bars) was performed for each FR and CDR region as described in Materials and methods. Differences between expected and observed values were assessed by Chi-squared test. Asterisks indicate significant difference (*P* < 0.01). (C) Comparison between expected and observed mutation frequencies targeting the WA/TW motifs in the FR and CDR regions. Analysis of expected (dark grey bars) and observed frequencies (grey bars) was performed for each FR and CDR region as described in Materials and methods. Differences between expected and observed values were assessed by Chi-squared test. Asterisks indicate significant difference (*P* < 0.01). (D) Mapping of the WRCY and its reverse RGYW motifs on the IGHV gene. The results were expressed as the number of mutations in each position. The most frequently mutated nucleotides were noted.

The motif RGYW and its reverse WRCY targeted by AID accounted for 47.3% of the mutated nucleotides, while mutation in WA or TW motifs targeted by DNA polymerases represented 32.7%. The frequency of mutations occurring in these motifs was strongly correlated with the percentage of mutations (*R*^2^ = 0.89, *P* < 0.001 for RGYW/WRCY motifs and *R*^2^ = 0.86, *P* < 0.001 for WA/TW motifs). Comparison of expected and observed frequencies in the RGYW/WRCY motifs or in the WA/TW motifs targeted by AID or polymerases, respectively, showed a significantly higher observed mutational frequency in these motifs than as a result of hazard (*P* < 0.001, Fig.4A).

Regarding the distribution of these mutated motifs along the IGHV gene, a higher frequency of mutations targeting the RGYW/WRCY and the WA/TW motifs was observed in CDR compared with FR regions (Fig.4B and C respectively). However, the observed higher frequency was because of a higher number of motifs in the CDR regions. Indeed, when comparing observed and expected frequencies according to the number of potential mutable motifs, a significant preference of mutations targeting RGYW/WRCY motifs could be observed in the FR regions and not in the CDR regions (Fig.4B). Analysis of motifs surrounding each mutation showed that nucleotide positions 9, 107, 119 and 275, preferentially targeted by mutations, were often localized in a RGWY or WRCY motif (Fig.4D).

### The characterization of BCR rearrangement pattern according to the age at CLL diagnosis suggests a tendency of increased replacement and transition-over-transversion mutations and a longer CDR3 length in older patients

As a decreased humoral immune diversity with ageing is well known, it has been suggested that age of appearance of CLL could have an impact on the characteristics of the BCR rearrangements patterns. To this aim, we stratified our cohort in three categories according to the age at diagnosis (See Materials and methods).

**Figure 5:**
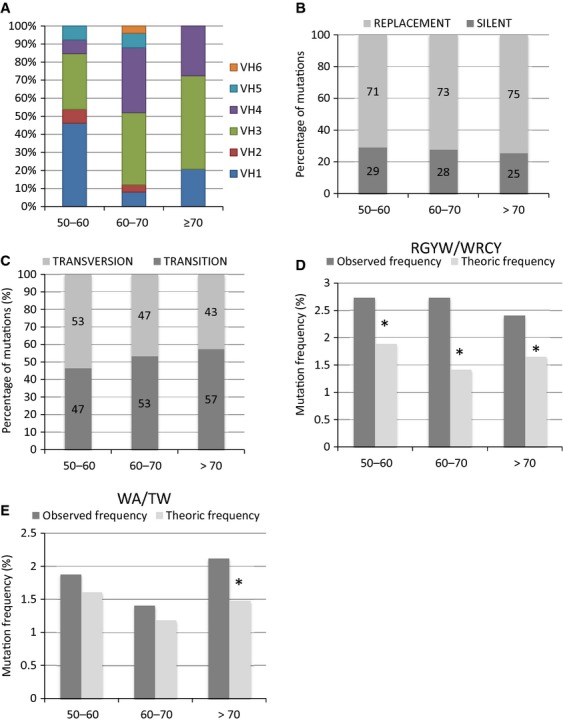
BCR rearrangements pattern related to the age at diagnosis of chronic lymphocytic leukaemia. (A) Comparison of IGHV family gene usage between each age categories. (B) Distribution of silent and replacement mutations according to the age categories. The results were expressed in percentage of mutations inducing an amino acid change (grey bars) or silent mutation (dark grey bars). (C) Distribution of transversions and transitions mutation according to the age categories. The results were expressed in percentage of mutations inducing a transversion (grey bars) or a transition (dark grey bars). (D) Comparison between expected and observed mutation frequencies targeting the RGYW/WRCY motifs according to the age categories. Analysis of expected or theoric (grey bars) and observed frequencies (dark grey bars) was performed for each age categories as described in Materials and methods. Differences between expected and observed values were assessed by Chi-squared test. Asterisks indicate significant difference (*P* < 0.01). (E) Comparison between expected and observed mutation frequencies targeting the WA/TW motifs according to age categories. Analysis of expected or theoric (grey bars) and observed frequencies (dark grey bars) was performed for each age categories as described in Materials and methods. Differences between expected and observed values were assessed by Chi-squared test. Asterisks indicate significant difference (*P* < 0.01).

First, we analysed the repartition of IGHV, IGHD and IGHJ genes in the three groups. The IGHV gene family usage was not statistically different between the age categories (Fig.5A). However, it is interesting to note that IGHV1 represented 50% of IGHV gene used in 50–60 years category of patients, whereas IGHV4 was uncommon in this age group. IGHV3 was the most observed gene family in older ages (patients >70 years). IGHD and IGHJ family usage did not significantly differ according to age (data not shown).

The repartition of patients between the mutated and the unmutated group did not significantly change with the age (Table4). Although the percentage of replacement mutation was increased in older individuals (Fig.5B), the percentage of patients with antigen-driving selection using the Lossos algorithm was similar in older and younger patients (Table4). The ratios of transition-over-transversion mutations were the same whatever the age of the patients (Fig.5C). The mutations targeting AID and polymerases motifs were also not significantly different between age categories (Table4). A significant greater proportion of mutations was observed in RGWY/WRCY motifs in all age categories (Fig.5D). However, the number of observed mutations in WA/TW motifs was significantly higher than expected only in older patients (Fig.5E). Concerning the CDR3 length, while no significant age-related differences appeared, a tendency of shorter CDR3 in older patients was noted (Table4). The decrease in CDR3 length with age could result from shorter IGHJ segments used and fewer N nucleotides added in 3′ of IGHD genes (Table4).

**Table 4:** Age-related clinical and biological characteristics, mean of mutations, mutational status, RGYW and WA targeting, Replacement/Silent ratio and CDR3 length

	50–60 years (*n* = 12)	60–70 years (*n* = 23)	>70 years (*n* = 21)	*P* value
Male (%)	7 (58.3%)	17 (73.9%)	13 (61.9%)	NS
Clinical characteristics				
Mean follow-up, *d* (range)	2198 (75–6286)	1419 (71–4367)	1049 (51–3284)	**0.05**
Stage at diagnosis*				
A (%)	9 (75%)	17 (74%)	16 (76.2%)	NS
B (%)	2 (16.7%)	4 (17.3%)	2 (9.5%)	
C (%)	1 (8.3%)	2 (8.7%)	3 (14.3%)	
Clinical course				
Stable (%)	5 (41.7%)	8 (34.4%)	14 (66.6%)	NS
Progressive (%)	7 (58.3%)	15 (63.6%)	7 (33.3%)	
Need of treatment				
Yes (%)	8 (66.6%)	18 (78.2%)	10 (50%)	NS
No (%)	4 (33.3%)	5 (21.7%)	10 (50%)	
Blood parameters				
Mean β2-microglobulin count, mg/l§	2.32	3.29	3.84	**0.02**
Mean haemoglobin count, g/dl	13.7	13.6	12.8	NS
Mean platelets count per μl	213.7	174	199	NS
Mean lymphocytes count per μl	30,724	41,885	15,249	NS
Mean LDH	356	315	312	NS
Mutations characteristics				
Mean of mutations	12.6	11.2	13.9	NS
Ratio mutated/unmutated status	6/12 (50%)	14/23 (61%)	13/21 (62%)	NS
Frequency of WA/TW motif targeted by mutation	1.9%	1.4%	2.1%	NS
Frequency of RGYW/WRCY motif targeted by mutation	2.7%	2.7%	2.4%	NS
R mutation scarcity in FR	3/8 (38%)	7/18 (39%)	5/20 (25%)	NS
R mutation excess in CDR	2/8 (25%)	5/18 (28%)	6/20 (30%)	NS
R mutation scarcity in FR and excess in CDR	2/8 (25%)	3/18 (17%)	3/20 (15%)	NS
CDR3 Components (mean length in bp)				
Total CDR3	59.5	53.7	52.3	NS
IGHV segment	9.9	9.5	9.2	NS
IGHD segment	15.5	13.2	15	NS
IGHJ segment	19.6	17.3	16.2	NS
P nucleotides	0.25	0.3	0.2	NS
N nucleotides in 5′ of IGHD	6.25	6.35	6	NS
N nucleotides in 3′ of IGHD	8	7.15	5.5	NS

* Binet classification stage at diagnosis.

§ β2-microglobulin count was obtained for 43 patients.

*P* values considered as significant (superior to 0.05) were noted in bold.

### There is no difference between clinical outcome and biological values according to age at CLL diagnosis

Clinical outcome were compared in the three age groups to evaluate whether age of diagnosis influences the disease stage and prognostic (Table4). There was no difference in stage at diagnosis, clinical course and treatment. The only parameter, which significantly differed between the age groups, was the mean follow-up. Concerning the biological values, there was no difference in the haemoglobin, the platelets and the lymphocytes count, and in the level of LDH according to the age at diagnosis. The β2-microglobulin was significantly increased with ageing.

## Discussion

We developed an in-house PCR protocol of multiplex using IGHV-Leaders primers. This method allowed the complete sequencing of the IGHV gene. The detection rate obtained with this method was comparable to those reported in series using the BIOMED-2 protocol [17,22]. The use of both primer sets slightly increased the detection rate of monoclonal rearrangements. Subsequently, we used this protocol for analysis of BCR rearrangements in our CLL cohort of patients.

The IGHV gene repartition between unmutated and mutated groups found in our study confirmed the biased usage of IGHV family in CLLs [5,8,15,16], while some differences in gene frequencies could be noticed. The frequencies of the IGHV genes used in BCR rearrangements in our cohort from the South of France were similar to those described in the Mediterranean and Northeastern region of Italy, but differed from the North of Europe [8,10]. This biased usage of IGHV family genes may reflect a geographical-dependent leukaemic repertoire as previously proposed [10]. Particularly, IGHV1-69 over-usage in the unmutated group of patients was consistent with previous observations in Northeastern region of Italy and Mediterranean populations [5,8,10]. The IGHV1-69/IGHD3-3/IGHJ6 rearrangement corresponding to a ‘stereotype’ CDR3 was described by Stamatopoulos *et al*. [11]. Regarding the CDR3 sequence, we also found the same motif consisting of YDFWSGYY in every patient. As previously described for Southern European CLLs, the IGHV3-21 gene frequently found in the North of Europe was observed in only two patients in our population [23].

Concerning IGHJ, previous studies observed an over-usage of IGHJ4 in CLL cells and preference for IGHJ4 in mutated status and IGHJ6 in unmutated group. In our study, IGHJ6 was more frequently used and we did not show a significant difference in the IGHJ genes repartition between mutated and unmutated groups.

Confirming previous studies, the CDR3 size was shorter in the mutated group than in the germline group [5,24]. We found that the CDR3 length was inversely correlated with the percentage of mutations. One hypothesis previously evoked in normal individuals by Rosner *et al*. is that CLL cells with shorter CDR3s have been selected for their higher affinity for antigen binding and subsequently undergoes hypermutation process [24].

Although we did not confirm a shorter CDR3 in IGHV3 segment as previously described [5], longer CDR3s were observed in IGHJ6 segment. We also confirmed that IGHV1-69 usage was associated with longer CDR3s. However, analysis of the contribution of each component of the CDR3 showed no significant influence of the IGHV length in the CDR3. Longer CDR3s in rearrangements with IGHV1-69 segment were more likely because of the ‘stereotype’ BCR, constituted by the IGHV1-69/IGHD3-3/IGHJ6 segments. Indeed, IGHD3-3 and IGHJ6 genes were significantly associated with longer CDR3 and participated in more nucleotides in the CDR3 length than other genes.

The mutational machinery responsible for SHM in normal B cells has been shown to introduce mutations in a non-random manner [25–27]. The features of canonical SHM are the targeting preference for RGYW motifs, nucleotide change bias for transitions and more frequent replacement mutations in CDR regions compared with FR. We found that the frequency of the mutations occurrence in the motifs was strongly correlated with the percentage of mutations, suggesting that AID and polymerases were the major contributors to the SHM process. Surprisingly, taking into account the number of RGWY motifs and the mutation rate in each region, we observed a preferential clustering of AID-induced mutations in the FR regions compared with CDRs. It is interesting to note that mapping of mutations into the IGHV gene indicated that FR3 region has a pattern similar to CDR regions with a higher mutation rate than FR1 and FR2 region. Moreover, these mutations are preferentially transitions as in CDR regions.

We also evaluated the characteristics of immunoglobulin rearrangement according to age at diagnosis. We found that the usage of IGHD and IGHJ genes was similar in all the age groups. These results are consistent with previous studies on healthy population [28,29]. Concerning IGHV usage, Wang *et al*. showed that the overall distribution of IGHV family usage was significantly different between older and younger normal individuals with a IGHV4 family predominance in elderly persons [29]. Such difference was not observed in our cohort of CLL patients. We observed a tendency to an augmentation of replacement and transition mutations, suggesting that malignant B-cell clones found in younger patients (<50 years) might carry less SHM than older patients. We also observed a progressive decrease in CDR3 size with age that could be as a result of a progressive decrease in the length of IGHV, IGHJ segments and in the number of nucleotides added in 3′ of IGHD. Altogether, these results suggested that malignant CLL cells from older patients derived from more mature cells than younger individuals and could reflect the level of chronic exposure to some environmental antigens. Concerning the biological and clinical parameters, the difference in the follow-up was because of the age categories. The increase in the β2-microgobulin with age was previously observed in healthy population [30].

In conclusion, this study showed that the features of CLL B cells in our cohort of patients followed those previously described in patients of Southern Europe origin and suggested a particular environmental antigenic pressure selection of B-CLL cells. These results allowed to unveil new findings regarding the molecular characterization of the B-CLL cells.
